# 

**Published:** 1883-07

**Authors:** 


### THE BRISTOL

### Medico-Chirurgical Journal.

A JOURNAL OF THE MEDICAL SCIENCES FOR THE
WEST OF ENGLAND AND SOUTH WALES.

*PUBLISHED UNDER THE AUSPICES OF*

*THE BRISTOL MEDICO-CHIRURGICAL SOCIETY.*

EDITED BY
J. GREIG SMITH,
M.A., F.R.S.E.

INDEXED S.G.O.

---

"Scire est nescire, nisi id me

Scire alius sciret."

---

SURGEON GEN'L'S OFFICE
LIBRARY.

*VOL. I.*

BRISTOL: J. W. ARROWSMITH;
LONDON: J. & A. CHURCHILL, 11 NEW BURLINGTON STREET.
1883.

WI
BR323

J.W. ARROWSMITH,

Printer and

Publisher,

QUAY STREET, BRISTOL

### CONTENTS OF VOL. I.

ORIGINAL PAPERS :— *Page.*

The Proofs of the Existence of a Phthisical Contagion. By R. SHINGLETON SMITH, M.D., B.Sc., M.R.C.P. ... ... 1

Clinical Evidence against the Contagiousness of Phthisis. By E. MARKHAM SKERRITT, M.D. Lond., B.S., B.A., M.R.C.P. ... ... ... ... ... ... ... ... ... ... ... 48

The Cardiograph in Medicine. By G. MUNRO SMITH, L.R.C.P. Lond., M.R.C.S. ... ... ... ... ... ... ... ... ... 71

Surgical Out-Patient Notes. By W. H. HARSANT, F.R.C.S. ... 82

Malarial Poisoning. By WM. JOHNSTON FYFFE, M.D. ... ... 143

A Record of Twenty-one Cases of Placenta Prævia. By JOSEPH GRIFFITHS SWAYNE, M.D. ... ... ... ... ... ... ... 166

An Epidemic of Tetanus. By ALFRED SHEEN, M.D. ... ... 173

A Study of "Medical Posture." By JOHN KENT SPENDER, M.D. ... ... ... ... ... ... ... ... ... ... ... ... 184

Abdominal Section in Perforating-Ulcer of the Stomach. A Suggestion. By NELSON C. DOBSON, F.R.C.S. ... ... 196

Retinoscopy. By ARTHUR B. PROWSE, M.D. ... ... ... ... 200

CLINICAL RECORDS :—

Two Cases of Compression of the Spinal Cord by Sarcomatous Growths from the Soft Membranes. By E. LONG FOX, M.D. Oxon., F.R.C.P. ... ... ... ... ... ... ... ... 100

A Case of Purulent Sub-Arachnitis, probably Metastatic. By GEORGE THOMPSON, M.D., L.R.C.P. ... ... ... ... ... 107

Cystic Neuroma of the Posterior Tibial Nerve. By J. FENTON EVANS, M.B. ... ... ... ... ... ... ... ... ... ... 109

Page.

Three Cases of Puerperal Convulsions treated by Venesection. By J. FULLER, M.K.Q.C.P. Irel. ... ... ... ... ... ... 111

Ulcerative Endocarditis of the Tricuspid Valve. Extensive Fibrinous Deposits. By J. E. SHAW, M.B. ... ... ... 114

Gastrostomy for Malignant Stricture of the Œsophagus. Hydatid Cyst of Liver. By the EDITOR ... ... ... ... ... ... ... 122

Recurrent Pendulous Fatty Tumour of Thigh. By the EDITOR 126

Primary Scirrhus of the Upper Jaw in a Child Two Years old 128

Case of Very Large Meningocele .. ... ... ... ... ... ... .. 129

Congenital Deformity of Right Leg and Left Hand ... ... ... 130

Cases of Muscular Atropy and Degeneration. By J. FENTON EVANS, M.B. ... ... .. ... ... ... ... ... ... ... ... 212

Note on the Utility of Iodoform in the Treatment of Phthisis, and its occasional Toxic Effects. By R. SHINGLETON SMITH, M.D., B.Sc., M.R.C.P. ... ... ... ... ... ... ... 218

A Case of Priapism. By A. H. BOYS, L.R.C.P. ... ... ... 224

Minute Hæmorrhages in the Pia Mater. By T. STEELE SHELDON, M.B. ... ... ... ... ... ... ... .. ... ... ... ... .. 225

Two Cases—One of Gouty Inflammation of the Testis, and another the Testicle of Mumps. By F. K. GREEN, F.R.C.S. ... ... ... ... ... ... ... ... ... ... ... ... ... 227

Successful Termination of a large Aneurism, without Surgical Interference. By HENRY WALDO, M.D. ... ... ... ... 232

UNIVERSITY COLLEGE, BRISTOL. MEDICAL SCHOOL:—

Address by DR. BEDDOE, F.R.S. ... ... ... ... ... ... ... 235

Prize List ... ... ... ... ... ... ... ... ... ... ... ... ... 245

NOTES ON BOOKS ... ... ... ... ... ... ... ... ... ... ... ... 131, 247

MEDICAL EXTRACTS ... ... ... ... ... ... ... ... ... ... ... 136, 253

INDEX ... ... ... ... ... ... ... ... ... ... ... ... ... ... 268

### PUBLICATIONS RECEIVED.—JULY, 1883.

*The Essentials of Bandaging, with Directions for Managing Fractures and Dislocations.* By BERKELEY HILL, M.B. Lond., F.R.C.S. Fifth Edition, revised and enlarged. London: Smith, Elder and Co. 1883.

*The Student's Manual of Venereal Diseases; being a Concise Description of those Affections and of their Treatment.* By BERKELEY HILL and ARTHUR COOPER. Third Edition. London: Smith, Elder and Co. 1883.

*Auscultation and Percussion, together with the other Methods of Physical Examination of the Chest.* By SAMUEL GEE, M.D. Third Edition. London: Smith, Elder and Co. 1883.

*A Guide to Therapeutics.* By ROBERT FARQUHARSON, M.P., M.D. Edin., F.R.C.P. Lond. Third Edition. London: Smith, Elder and Co. 1883.

*Hospital Mortality.* By H. BURDETT. London: Smith, Elder and Co. 1883.

*Observations on Lithotomy, Lithotrity, and the Early Detection of Stone in the Bladder, with a Description of a New Method of Tapping the Bladder.* By REGINALD HARRISON, F.R.C.S. London: J. and A. Churchill. 1883.

*Birmingham Medical Review.* Vol. XIII., No. 58. June, 1883.

### *Bristol Medico-Chirurgical Journal.*

### TERMS FOR ADVERTISEMENTS.

| | | | | Special Places. | | | Outside of Cover. | | |
|---|---|---|---|---|---|---|---|---|---|
| | £ | s. | d. | £ | s. | d. | £ | s. | d. |
| WHOLE PAGE ... | 1 | 1 | 0 | 1 | 11 | 6 | 2 | 2 | 0 |
| HALF PAGE ... ... | 0 | 12 | 6 | 0 | 17 | 6 | 1 | 5 | 0 |
| QUARTER PAGE ... | 0 | 7 | 6 | 0 | 10 | 0 | 0 | 15 | 0 |

INSETS (single leaf), 5/-.

The next number of the Journal will be published in December, 1883.

Orders for Advertisements should be sent to the Publication Secretary,

L. M. GRIFFITHS, 9 Gordon Road, Clifton.

### Past Presidents

OF THE

### Bristol Medico-Chirurgical Society.

---

1874—75. FREDERICK BRITTAN, M.D., B.A. Dub.

1875—76. ROBERT WILLIAM COE.

1876—77. SAMUEL MARTYN, M.D. Ed.

1877—78. AUGUSTIN PRICHARD, M.D. Berlin.

1878—79. HENRY EDWARD FRIPP, M.D. Würzburg.

1879—80. WILLIAM MICHELL CLARKE.

1880—81. JOSEPH GRIFFITHS SWAYNE, M.D. Lond.

1881—82. EDWARD LONG FOX, M.D. Oxon.

### Bristol Medico-Chirurgical Society.

---

### President.

JAMES G. DAVEY, M.D. St. And.

### President-Elect.

W. JOHNSTONE FYFFE, M.D. Dub.

### Honorary Secretary.

R. SHINGLETON SMITH, M.D., B.Sc. Lond.

### Committee.

*Past Presidents.*

R. W. COE.

AUGUSTIN PRICHARD, M.D. Berlin.

W. MICHELL CLARKE.

J. G. SWAYNE, M.D. Lond.

E. LONG FOX, M.D. Oxon.

NELSON C. DOBSON.
L. M. GRIFFITHS.
F. POOLE LANSDOWN.
E. MARKHAM SKERRITT, M.D., B.S., B.A. Lond.
J. GREIG SMITH, M.B., C.M., M.A. Aberd., F.R.S.E.
W. H. SPENCER, M.D., M.A. Cantab.

---

Medical Men wishing to join the Society are requested to communicate with the *Honorary Secretary*, 9 Richmond Hill, Clifton. *The Annual Subscription is 10/6.*

---

*Extracts from Journal By-laws.*

4.—The *Journal* shall be delivered free to Subscribers, and also to Members of the Society whose subscriptions are not in arrear.

6.—All Subscriptions for the *Journal* shall be paid in advance annually.

---

Communications intended for insertion in the *Journal*, Books for Review, &c., should be sent to the Editor, J. Greig Smith, M.A., F.R.S.E., 16 Victoria Square, Clifton.

Authors requiring Reprints of their Papers should communicate with the Publisher.

### LIST OF SUBSCRIBERS

TO THE

### Bristol Medico-Chirurgical Journal.

*Those marked * are Members of the Society.*

Communications referring to the delivery of the *Journal* should be sent to the Publication Secretary, L. M. Griffiths, 9 Gordon Road, Clifton, to whom Subscriptions are to be paid *in advance*, and to whom information of any inaccuracy in this list should be given.

The Subscription to the Journal for 1883 is 5/-.

| *Name.* | *Residence.* |
|---|---|
| Ackland, W. H., M.D. St. And. | Bideford. |
| Adams, George | 5 Oakfield Road, Clifton. |
| Adams, James, M.D. Aberd. | Ashburton. |
| Alexander, James, M.D., M.Ch. Dub. | Paignton. |
| Alford, Richard | 6 Oriel Terrace, Weston-super-Mare. |
| *Andrews, Onslow, M.D. St. And. | 159 White Ladies Road, Bristol. |
| Anstie, Thomas B. | 21 Northgate Street, Devizes. |
| *Atchley, George F., M.B. Lond. | Tyndall's Park, Bristol. |
| Baron, B. J., M.B., C.M. Ed. | 12 Richmond Hill, Clifton. |
| Batten, Rayner W., M.D. Lond. | 1 Brunswick Square, Gloucester. |
| *Beddoe, John, M.D. Ed., B.A. Lond., F.R.S. | Mortimer House, Clifton. |
| Bennett, W. S. | 12 The Terrace, Penzance. |
| Berry, F. C., M.D., B.Ch. Dub. | Lynton. |
| *Blacker, A. E. | 7 Unity Street, Bristol. |
| Blacker, E. | Midsomer Norton. |
| Blaxall, F. H., M.D. St. And. | Clan Lodge, Bath. |
| Blomfield, Arthur G., M.D. Aberd. | Devon and Exeter Hospital, Exeter. |
| *Board, Edmund C. | 7 Caledonia Place, Clifton. |
| Bond, Francis T., M.D. Lond. | 1 Beaufort Buildings, Gloucester. |
| *Bousfield, E. C. | Ashley Road, Bristol. |
| Bower, Ernest D. | 1 Norfolk Terrace, Gloucester. |
| Boyle, Thomas | Newquay. |
| *Boys, Arthur Henry | Lodway villa, Pill. |
| Bridgman, I. T. | Berkeley. |
| Broom, John, M.D. Brussels | St. Paul's Road, Clifton. |
| Broster, Alfred, M.D. Giessen | 6 Brunswick Square, Bristol. |
| *Burder, George F., M.D. Aberd. | 63 Oakfield Road, Clifton. |
| Burroughs, E. F. H. | Ridgway, Frome. |
| Bush, J. Paul | Royal Infirmary, Bristol. |
| Cann, Francis M. | Sefton House, Dawlish. |
| Carey, J., M.D. Lond. | 2 Hamdon Villas, Taunton. |
| *Carr, Alexander | Wells Road, Bristol. |

| | |
|---|---|
| *Carter, W. F. | Coronation Road, Bristol. |
| *Challacombe, J. P., M.D. Ed. | 45 Redland Road, Bristol. |
| Clark, Thomas | Dunster. |
| *Clark, T. E., M.D., C.M. Aberd. | 3 Victoria Square, Clifton. |
| Clarke, Fred. H. | Kingsbridge. |
| *Clarke, W. Michell | 2 York Buildings, Clifton. |
| Clay, R. Hogarth, M.D. Ed. | 4 Windsor Villas, Plymouth. |
| Coathupe, Edwin W. | 7 Clifton Park, Clifton. |
| Cockey, Edmund | West Lodge, Frome. |
| *Coe, R. W. | 14 Berkeley Square, Bristol. |
| Coker, T. V. | 2 Chesterfield Place, Clifton. |
| Cole, Thomas, M.D. Lond. | 17 Paragon, Bath. |
| *Collins, H. W. | Wrington. |
| Colman, Thomas J., M.D. Ed. | Redland Road, Bristol. |
| Colmer, Ptolemy S. H. | Yeovil. |
| Colthurst, J. B. | Wincanton. |
| Cooke, A. S. | Badbrook House, Stroud. |
| Cooper, Herbert | Hampstead, London, N.W. |
| *Corbould, George G. | 22 Berkeley Square, Bristol. |
| Couch, James | Christina Street, Swansea. |
| Cripps, Edward | Cirencester. |
| *Crisp, Nathaniel | Chandos House, Keynsham. |
| *Cross, F. Richardson, M.B. Lond. | Chandos Villa, Clifton. |
| *Crossman, Edward | Hambrook. |
| Cunningham, A. G., M.B., C.M. Aberd. | Stapleton Road, Bristol. |
| Daly, George H., M.D. St. And. | Burley House, Chippenham. |
| *Davey, James G., M.D. St. And. | 4 Redland Park, Bristol. |
| *Davies, D. S., M.B. Lond. | 41 Queen Square, Bristol. |
| Davis, Theodore, M.D. Lond. | Beachcroft, Clevedon. |
| Dening, Edwin | Stow-on-the-Wold. |
| *Dew, Henry R. | 25 Berkeley Square, Bristol. |
| *Dobson, Nelson C. | 27 Victoria Square, Clifton. |
| *Dowson, C. H. | Tyndall's Park, Bristol. |
| Doyne, R. W. | Fishponds. |
| Dunbar, Eliza L. Walker, M.D. Zürich | 9 Oakfield Road, Clifton. |
| Eadon, W. F. Bailey | Hambrook. |
| *Eager, Reginald, M.D. Lond. | Northwoods. |
| Edgeworth, T. F. | 1 Stokes Croft, Bristol. |
| *Elliott, Christopher, M.D. Dub. | 155 White Ladies Road, Bristol. |
| *Elwin, J. Fountain | 17 Clyde Road, Bristol. |
| *Evans, J. Fenton, M.B., C.M. Ed. | 10 Pembroke Road, Clifton. |
| *Ewens, John | 20 Cotham New Road, Bristol. |
| Fairbanks, W., M.D., C.M. Ed. | Wells. |
| *Fendick, R. George | 3 Claremont Place, Clifton. |
| Fernie, Andrew, M.D. Dur. | Barnstaple. |
| Fernie, James | Stratton St. Margaret. |
| Ferris & Co. | Union Street, Bristol. |
| Fiddian, Alexander P., M.B. Lond. | 6 Brighton Terrace, Cardiff. |
| Fletcher, Henry Studd | Lydbrook. |
| Forty, D. H. | Wotton-under-Edge. |
| Fox, Arthur E. W., M.B., C.M. Ed. | 16 Gay Street, Bath. |
| *Fox, Bonville B., M.B., M.A. Oxon. | Brislington. |
| *Fox, E. Long, M.D. Oxon. | Church House, Clifton. |
| Fry, J. Farrant | 203 St. Helen's Road, Swansea |

| | |
|---|---|
| Fullerton, R., M.B., B.Ch. Dub. ... ... ... | City and County Asylum, Stapleton. |
| *Fyffe, W. Johnstone, M.D. Dub.... ... ... | 2 Rodney Place, Clifton. |
| Gardiner, George ... ... ... ... ... ... | 1 Redcliff Hill, Bristol. |
| *Gibbs, Alfred N. Godby ... ... ... ... ... | Children's Hospital, Bristol. |
| *Gloag, G. A. ... ... ... ... ... ... ... ... ... | 6 College Green, Bristol. |
| Gooding, J. Callender, M.D. Ed. ... ... | Berkeley Street, Cheltenham. |
| Goss, T. Biddulph ... ... ... ... ... ... | 36 Paragon, Bath. |
| Gowing, B. C. ... ... ... ... ... ... ... ... | Avon House, Salisbury. |
| Grace, Edward Mills ... ... ... ... ... | Park House, Thornbury. |
| Grace, Henry ... ... ... ... ... ... ... ... ... | Kingswood. |
| Grace, W. G. ... ... ... ... ... ... ... ... ... | 57 Stapleton Road, Bristol. |
| Green, F. K. ... ... ... ... ... ... ... ... ... | 3 Gay Street, Bath. |
| Gregory, George S. ... ... ... ... ... ... | The Green, Stroud. |
| *Griffiths, L. M. ... ... ... ... ... ... ... | 9 Gordon Road, Clifton. |
| Grote, G. W. ... ... ... ... ... ... ... ... | 32 Portland Square, Bristol. |
| Hardyman, Charles E. ... ... ... ... ... | 24 Crockherbtown, Cardiff. |
| Harper, Joseph ... ... ... ... ... ... ... | Bear Street, Barnstaple. |
| *Harrison, A. J., M.B. Lond. ... ... ... ... | Guthrie Road, Clifton. |
| *Harsant, W. H. ... ... ... ... ... ... ... | 16 Pembroke Road, Clifton. |
| Hart, Gratian C. Barry ... ... ... ... ... | The Shrubbery, Weston-super-Mare. |
| Hayman, A. G.... ... ... ... ... ... ... ... | 1 Pembroke Road, Clifton. |
| *Heaven, John C. ... ... ... ... ... ... ... | Avonmouth. |
| *Herapath, C. K. C. ... ... ... ... ... ... | 11 Brunswick Square, Bristol. |
| *Hetling, H. E. ... ... ... ... ... ... ... ... | Stapleton Road, Bristol. |
| Hichens, James S.... ... ... ... ... ... ... | Redruth. |
| Hickes, Thomas ... ... ... ... ... ... ... | St. Michael's Home, Cheddar. |
| Hine, S. D. ... ... ... ... ... ... ... ... ... | Ilminster. |
| Hinton, Joseph ... ... ... ... ... ... ... | Warminster. |
| Hopkins, H. Culliford ... ... ... ... ... | 4 Gay Street, Bath. |
| Horder, T. Garrett ... ... ... ... ... ... | 32 Crockherbtown, Cardiff. |
| Howse, William ... ... ... ... ... ... ... | London Street, New Swindon. |
| Hudson, Robert S., M.D., M.Ch. Qu. Univ. I. | 10 Trewirgie Road, Redruth. |
| Hyatt, Brownlow N.... ... ... ... ... ... | Park View, Shepton Mallet. |
| *Imlay, G. A., M.B., C.M. Aberd. ... ... ... | 4 Apsley Villas, Bristol. |
| Infirmary, The Royal ... ... ... ... ... ... | Bristol. |
| *Isaac, G. W., M.B., C.M. Aberd. ... ... ... | 49 White Ladies Road, Bristol. |
| Jenkins, John Henry ... ... ... ... ... ... | Liskeard. |
| Jones, Evan ... ... ... ... ... ... ... ... | Ty-mawr, Aberdare. |
| Joynes, Francis James ... ... ... ... ... | Eagle House, Dursley. |
| *Keall, W. P. ... ... ... ... ... ... ... ... | Coronation Road, Bristol. |
| Kempe, Arthur W. ... ... ... ... ... ... | 20 St. Sidwell's, Exeter. |
| *Kendall, Bernard ... ... ... ... ... ... | 3 Downside Road, Clifton. |
| Kinneir, R. ... ... ... ... ... ... ... ... ... | The Tower House, Malmesbury. |
| Kirkman, J. Miller ... ... ... ... ... ... | Wootton Bassett. |
| Laing, W. A. Gordon, M.B., C.M. Glasg. ... | Mevagissey. |
| *Lansdown, F. Poole ... ... ... ... ... ... | 19 White Ladies Road, Clifton. |
| Latimer, H. A. ... ... ... ... ... ... ... ... | 72 Mansel Terrace, Swansea. |
| *Lawrence, A. E. Aust, M.D., C.M. Aberd. | 15 Richmond Hill, Clifton. |
| Lawrence, A. G., M.D. St. And. ... ... ... | Chepstow. |
| *Lawrence, Henry ... ... ... ... ... ... ... | 9 Park Row, Bristol. |
| *Laxton, Henry ... ... ... ... ... ... ... | 12 Apsley Road, Clifton. |
| *Lewis, J. R., M.B., C.M. Glasg. ... ... ... | 20 Gloucester Road, Bristol. |

Liddon, William, M.B. Lond. ... ... ... Church Square, Taunton.

Lloyd, Walter E. ... ... ... ... ... ... ... 60 York Road, Bristol.

Lodge, John ... ... ... ... ... ... ... ... Keynsham.

Logan, Frederic T. B. ... ... ... ... ... ... Dean Lane, Bristol.

Lovell, W. Day ... ... ... ... ... ... ... The Abbey, Bradford-on-Avon.

Lovell, William Frederick ... ... ... ... Compton Martin.

*Luffman, W. C. ... ... ... ... ... ... ... 8 Cotham New Road, Bristol.

*Lyons, A. de Courcy, M.B., C.M. Aberd. ... Henley Lodge, Yatton.

*Macintire, J. H. Lee ... ... ... ... ... ... Royal Infirmary, Bristol.

Maclean, J. Campbell, M.B., C.M. Ed. ... Swindon House, Swindon.

Magrath, J. A., M.D. Glasg. ... ... ... ... 7 Den Crescent, Teignmouth.

Manning, H. J. ... ... ... ... ... ... ... ... Laverstock House, Salisbury.

*Marchant, W. R. F., M.D. St. And. ... ... 91 White Ladies Road, Bristol.

Marley, Henry F. ... ... ... ... ... ... ... The Nook, Padstow.

Marsh, Charles J. ... ... ... ... ... ... ... Yeovil.

*Marshall, Henry, M.D. Ed., F.R.S.E. ... 28 Caledonia Place, Clifton.

Mason, Frederick ... ... ... ... ... ... ... 20 Belmont, Bath.

Mason, S. B. ... ... ... ... ... ... ... ... ... 12 Park Terrace, Pontypool.

Mason, William ... ... ... ... ... ... ... ... St. Austell.

Matthews, Charles E. ... ... ... ... ... ... Stapleton Road, Bristol.

Mayor, T. O. ... ... ... ... ... ... ... ... ... 40 Apsley Road, Clifton.

Meredith, John, M.D. Ed. ... ... ... ... Wellington.

Milne, John, M.B., C.M. Aberd. ... ... ... 97 Clarence Road, Bristol.

Milward, James ... ... ... ... ... ... ... ... 54 Charles Street, Cardiff.

Morgan, E. Rice ... ... ... ... ... ... ... ... Morriston, Swansea.

Morgan, John ... ... ... ... ... ... ... ... ... Langport.

Morgan, William, M.D. Aberd. ... ... ... Dumfries Place, Cardiff.

Moynan, W. Arthur, M.D., M.Ch. Qu. Univ. I. Somerset County Asylum, Wells.

Murray, William, M.B., C.M. Ed. ... ... Staple Hill.

Napier, T. W. A., M.B., C.M. Aberd. ... ... Strathwye, Tintern.

Needham, Frederick, M.D. St. And. ... ... Barnwood House, Gloucester.

*Newman, Charles ... ... ... ... ... ... ... 19 Portland Square, Bristol.

Newton, Charles John ... ... ... ... ... ... Oriel Lodge, Cheltenham.

Nicholson, T. D., M.D., C.M. Ed. ... ... 2 White Ladies Road, Clifton.

Norman, J. H. ... ... ... ... ... ... ... ... ... The Clotworthy, Winkleigh.

Norman, J. W.... ... ... ... ... ... ... ... ... Edde Cross House, Ross.

*Norton, J. A., M.D., C.M. Aberd. ... ... ... 8 Redcliff Hill, Bristol.

*Norton, T. Chalmers ... ... ... ... ... ... 12 King Square, Bristol.

Nourse, W. J. Chichele ... ... ... ... ... ... Morchard Bishop.

Ollerhead, T. J. ... ... ... ... ... ... ... ... Hillbury, Minehead.

*Ormerod, Henry ... ... ... ... ... ... ... ... Westbury-upon-Trym.

Padley, George ... ... ... ... ... ... ... ... 2 Northampton Terrace, Swansea.

*Parette, James ... ... ... ... ... ... ... ... 16 College Green, Bristol.

Parry, J. H. ... ... ... ... ... ... ... ... ... 99 Ashley Road, Bristol.

*Parson, T. Cooke ... ... ... ... ... ... ... 21 White Ladies Road, Bristol.

Parsons, Francis J. C. ... ... ... ... ... ... King Square, Bridgwater.

Parsons, J. D. F., M.D. St. And. ... ... ... 13 Dowry Square, Clifton.

Partridge, Thomas ... ... ... ... ... ... ... Stroud.

Pauli, G. C. P. ... ... ... ... ... ... ... ... 240 York Road, Bristol.

Pearce, Edward T. ... ... ... ... ... ... ... Holsworthy.

Pearse, Fred. E., M.D. St. And. ... ... ... Hall House, Frome.

*Penny, W. J. ... ... ... ... ... ... ... ... ... General Hospital, Bristol.

Penruddocke, Charles ... ... ... ... ... ... Winchcombe.

| | |
|---|---|
| Perkins, E. B. Steele ... ... ... ... ... | 21 Magdalen Street, Exeter. |
| Perkins —., M.D. New York ... ... ... ... | 14 Victoria Square, Clifton. |
| Perrin, J. D. ... ... ... ... ... ... ... ... ... | Temple Cloud. |
| Phelps, W. Harford G., M.D. Aberd. ... | Weston-super-Mare. |
| Phelps, William H. ... ... ... ... ... ... ... | 123 Ashley Road, Bristol. |
| Philipps, S. Rees, M.D., M.Ch. Qu. Univ. I. | Wonford House, Exeter. |
| Phillips, Edward E. ... ... ... ... ... ... ... | Broadwater House, Southend. |
| *Pickering, C. F. ... ... ... ... ... ... ... ... | St. Paul's Road, Clifton. |
| *Pigeon, H.... ... ... ... ... ... ... ... ... ... | 15 White Ladies Road, Clifton. |
| Pizey, G. F. P.... ... ... ... ... ... ... ... | Rydal Villa, Clevedon. |
| Porter, P.... ... ... ... ... ... ... ... ... ... | 109 Fore Street, Saltash. |
| Potter, Samuel R., M.B., B.A. Dub. ... ... | Collumpton. |
| Powell, William, M.B. Lond. ... ... ... | Hill Garden, Torquay. |
| Prankerd, John ... ... ... ... ... ... ... ... | Langport. |
| *Prichard, Arthur W.... ... ... ... ... ... ... | 31 St. Paul's Road, Clifton. |
| *Prichard, Augustin, M.D. Berlin ... ... ... | 4 Chesterfield Place, Clifton. |
| *Prichard, J. E., M.B., B.A. Oxon. ... ... | 243 Cheltenham Road, Bristol. |
| Prideaux, T. Engledue P. ... ... ... ... ... | Wellington. |
| Prowse, Arthur B., M.D. Lond. ... ... ... | 4 Rodney Cottages, Clifton. |
| Randolph, Charles ... ... ... ... ... ... ... | Milverton. |
| Rogers, George, M.D. St. And. ... ... ... | 6 Portland Square, Bristol. |
| Rossiter, George F., M.B. Lond. ... ... | Cairo Lodge, Weston-super-Mare. |
| *Roué, W. Barrett, M.D., M.S. Dur. ... ... | 165 White Ladies Road, Bristol. |
| Roxburgh, Robert, M.B. Ed. ... ... ... ... | 1 Victoria Buildings, Weston-super-Mare. |
| *Rudge, C. King ... ... ... ... ... ... ... ... | 145 White Ladies Road, Bristol. |
| Russell, M. W. H. ... ... ... ... ... ... ... | Royal United Hospital, Bath. |
| Salmon, W. G. ... ... ... ... ... ... ... ... | Thornbury. |
| Scott, James, M.B., C.M. Ed.... ... ... ... | 7 St. Mary's Vale, Chatham. |
| Scott, Richard J. H. ... ... ... ... ... ... ... | 13 Bladud Buildings, Bath. |
| Searle, George C. ... ... ... ... ... ... ... | Brixham. |
| Seward, W. J., M.B. Lond. ... ... ... ... | County Asylum, Colney Hatch, Lond., W. |
| Sewart, J. H. ... ... ... ... ... ... ... ... ... | Lostwithiel. |
| Shapter, Lewis, M.D. Cantab. ... ... ... | The Barnfield, Exeter. |
| *Shaw, J. E., M.B., C.M. Ed. ... ... ... ... | 11 Lansdown Place, Clifton. |
| Sheen, A., M.D. St. And. ... ... ... ... ... | 23 Newport Road, Cardiff. |
| Sheldon, T. Steele, M.B. Lond.... ... ... | Somerset County Asylum, Wells. |
| *Shorland, Walter E. ... ... ... ... ... ... | 211 Cheltenham Road, Bristol. |
| Skeate, Edwin ... ... ... ... ... ... ... ... | 16 Paragon, Bath. |
| *Skelton, Henry ... ... ... ... ... ... ... | Downend. |
| *Skerritt, E. Markham, M.D., B.S., B.A. Lond. | Richmond Hill, Clifton. |
| *Skinner, Stephen, M.B., C.M. Aberd. ... | Ferndale, Clevedon. |
| Smith, George ... ... ... ... ... ... ... ... | Axbridge. |
| *Smith, George ... ... ... ... ... ... ... ... | The Coombe, Westbury-upon-Trym. |
| *Smith, G. Munro ... ... ... ... ... ... ... ... | 70 Pembroke Road, Clifton. |
| *Smith, J. Greig, M.B., C.M., M.A. Ab., F.R.S.E. | 16 Victoria Square, Clifton. |
| Smith, J. Sidney, M.D. St. And. ... ... ... | Tiverton. |
| *Smith, R. Shingleton, M.D., B.Sc. Lond. | 9 Richmond Hill, Clifton. |
| *Smith, Samuel ... ... ... ... ... ... ... ... | 7 Kingsdown Parade, Bristol. |
| Society, Plymouth Medical (*Hon. Sec.* Dr. | A. H. Bampton). |
| Somer, James ... ... ... ... ... ... ... ... ... | Broadclyst. |
| *Spencer, W. H., M.D., M.A. Cantab. ... ... | 5 Lansdown Place, Clifton. |
| Spender, John K., M.D. Lond. ... ... ... | 17 Circus, Bath. |
| Stansfeld, G. M. ... ... ... ... ... ... ... ... | 19 Victoria Square, Clifton. |
| Steel, S. H., M.B. Lond. ... ... ... ... ... | Abergavenny. |

| | |
|---|---|
| *Steele, Charles, M.D. Dur. ... ... ... ... ... | 17 Richmond Hill, Clifton. |
| *Steven, Alexander, M.D., C.M. Ed. ... ... | 6 West Mall, Clifton. |
| *Stevens, Frederick G. ... ... ... ... ... ... | 180 Stapleton Road, Bristol. |
| *Stewart, James, B.A. Qu. Univ. I. ... ... | Sneyd Park, Bristol. |
| Stock, G. ... ... ... ... ... ... ... ... ... ... | Barton Hill Road, Bristol. |
| Storry, Frederick W. ... ... ... ... ... ... | Stroud. |
| Swain, W. Paul ... ... ... ... ... ... ... ... | 17 The Crescent, Plymouth. |
| *Swayne, J. G., M.D. Lond. ... ... ... ... | 74 Pembroke Road, Clifton. |
| *Swayne, S. H.... ... ... ... ... ... ... ... ... | 129 Pembroke Road, Clifton. |
| Sydney-Turner, A. M. ... ... ... ... ... | College Green, Gloucester. |
| Tayler, Geo. Christopher, M.D. Lond. ... | Trowbridge. |
| Taylor, Frank ... ... ... ... ... ... ... ... | 5 Colston's Parade, Bristol. |
| *Taylor, J. ... ... ... ... ... ... ... ... ... | 5 Ashley Road, Bristol. |
| Taylor, Thomas Henry ... ... ... ... ... | Prospect House, Thornbury. |
| Thomas, A. Garrod, M.D., C.M. Ed. ... ... | 28 Bridge Street, Newport, Mon. |
| Thomas, R. W. ... ... ... ... ... ... ... ... | Tregare House, Keynsham. |
| *Thompson, George, M.D. Dur. ... ... ... | City and County Asylum, Stapleton. |
| Thomson, G. J. C., M.B. Dur.... ... ... ... | Rook Lane House, Frome. |
| Thomson, G. S., M.D. Ed. ... ... ... ... | 8 College Road, Clifton. |
| Thomson, Spencer, M.D. St. And. ... ... | Ashton, Devon. |
| *Tivy, W. J. ... ... ... ... ... ... ... ... ... | 8 Lansdown Place, Clifton. |
| Todd, W. J. ... ... ... ... ... ... ... ... ... | North Petherton. |
| Tosswill, Louis H., M.B. Cantab. ... ... | 49 Magdalen Street, Exeter. |
| Twining, A. H. ... ... ... ... ... ... ... ... | Salcombe. |
| Tyley, R. P., M.D. St. And. ... ... ... ... | Wedmore. |
| Valentine, Edmund W. ... ... ... ... ... | Somerton. |
| Wade, A. Law, M.D. Dub. ... ... ... ... | Somerset County Asylum, Wells. |
| *Waldo, Henry, M.D., C.M. Aberd. ... ... | 19 Pembroke Road, Clifton. |
| Walsh, David H. ... ... ... ... ... ... ... ... | Sneyd Park, Bristol. |
| Walter, William Henry ... ... ... ... ... | Knapp Villa, South Petherton. |
| Ward, J. L. W. ... ... ... ... ... ... ... ... | Victoria Street, Merthyr Tydfil. |
| *Wathen, J. Hancocke ... ... ... ... ... | 18 Richmond Hill, Clifton. |
| Watson, J. Adam ... ... ... ... ... ... ... | Chudleigh. |
| Watters, George T. B., M.D., C.M. Ed. ... | Stonehouse. |
| Waugh, Alexander ... ... ... ... ... ... | Midsomer Norton. |
| *Weatherly, Lionel A., M.D., C.M. Aberd. | Portishead. |
| Webb, W. H. ... ... ... ... ... ... ... ... ... | Kingsbridge. |
| *Webster, Thomas ... ... ... ... ... ... | Redland Hill, Bristol. |
| *Welsh, F. F. ... ... ... ... ... ... ... ... ... | 1 Upper Belgrave Road, Clifton. |
| Wickham, W. ... ... ... ... ... ... ... ... ... | Hill House, Tetbury. |
| Wicksteed, Francis W. S. ... ... ... ... | Chester House, Weston-super-Mare. |
| Willett, M., M.D. St. And. ... ... ... ... | 87 Ashley Road, Bristol. |
| Williams, Eubulus, M.D. St. And. ... ... | 1 Lansdown Place, Clifton. |
| Williams, William Henry, M.D. Lond. ... | Sherborne. |
| Wilton, John P. ... ... ... ... ... ... ... | 10 College Green, Gloucester. |
| Winterbotham, L. ... ... ... ... ... ... | Bays Hill, Cheltenham. |
| Wise, N. V. ... ... ... ... ... ... ... ... ... | Fore Street, Trowbridge. |
| Workman, C. J., M.D. Ed. ... ... ... ... | Teignmouth. |

